# Multimodal Pain Management in Knee Osteoarthritis: A Comparative Study of Pregabalin and Duloxetine as Adjuncts to Naproxen

**DOI:** 10.7759/cureus.86076

**Published:** 2025-06-15

**Authors:** Muhammad Nauman Zafar, Syeda Fatima, Saima Ambreen, Muhammad Khurram, Tahir Iqbal, Muhammad Arif, Afzaal Aleem Khan

**Affiliations:** 1 Medicine, Holy Family Hospital, Rawalpindi, PAK; 2 Medicine, Shifa Tameer-e-Millat University, Shifa College of Medicine, Islamabad, PAK

**Keywords:** depression, effectiveness, knee osteoarthritis, pain, sleep quality

## Abstract

Objective: This single-blinded, randomised, prospective study was carried out to investigate the effectiveness of a combination drug regimen including pregabalin and duloxetine as adjuncts to naproxen in pain management of patients with knee osteoarthritis (OA).

Methods: One hundred and five patients were inducted into the study following the inclusion and exclusion criteria. Using simple randomisation, patients were allocated into group A (naproxen only), group B (naproxen + duloxetine), and group C (naproxen + pregabalin). Visual analogue scale (VAS) and Western Ontario and McMaster Universities Osteoarthritis Index (WOMAC) scores were used to measure pain severity, while secondary outcomes of sleep quality and depression were assessed by using the Pittsburgh Sleep Quality Index (PSQI) and Beck Depression Inventory (BDI) scale, respectively. Assessments were done at day 0, week 4, and week 12 in the study. Appropriate tests were utilised to analyse the data.

Results: Mean pain scores at 12 weeks were significantly lower in group B (p=0.009 for VAS, p=0.002 for WOMAC) and group C (p=0.012 for VAS, p=0.005 for WOMAC), as compared to group A (naproxen only). Sleep quality (p=0.00) and depression scales (p=0.00) were also similarly improved in the combination drug regimen groups as compared to the naproxen-only group.

Conclusion: Addition of either duloxetine or pregabalin to the naproxen drug regimen may result in better management and improved quality of life in patients suffering from knee OA.

## Introduction

Osteoarthritis (OA) is a persistent, progressive and degenerative joint condition commonly found in middle-aged and older adults [1]. Age, gender, obesity and unfavourable mechanical conditions are the principal risk factors contributing to OA [2]. Radiological features favouring OA include joint space narrowing and the presence of osteophytes [3]. Pain from OA is known to be a hybrid type of pain, combining nociceptive as well as neuropathic elements [4]. During the initial stages of OA, pain usually arises secondary to activity; however, with disease progression, it tends to become constant and intense. It has been postulated that central sensitisation has a role in the chronic pain of OA, due to prolonged activation of spinal and supraspinal neurons [5].

Duloxetine is a serotonin and norepinephrine uptake inhibitor, which possesses anti-depressant, anxiolytic and central pain inhibitory properties [6]. Due to its influence on the descending pain pathways, it has been postulated to be beneficial in chronic musculoskeletal pain, including pain arising from OA [7].

Pregabalin is a neuropathic agent that has a role in pain management secondary to central neuronal hypersensitivity. It attaches to the voltage gated calcium channels and blocks the release of neurotransmitters. Pregabalin possesses a huge potential for meeting analgesic demands due to its effectiveness in neuropathic conditions, and a subgroup of osteoarthritic patients report neuropathic aspects to their pain [8].

Non-steroidal anti-inflammatory drugs (NSAIDs) have been in use for OA for a long time. Naproxen and ibuprofen have been used in OA for many years. Specifically, naproxen offers significantly more pain control as compared to placebo and ibuprofen and improves nocturnal discomfort [9]. However, prolonged use of NSAIDs is usually discouraged due to their adverse effects on cardiovascular, gastrointestinal and renal systems [10].

A study carried out in our local community compared NSAIDs with duloxetine for knee OA, which showed a superior role of duloxetine (mean visual analogue scale (VAS) score of 3.56 versus 4.45) [11]. Another study proved significant improvement in pain scores by using pregabalin in OA [12]. Our study is a single-blinded, randomised, prospective study which aimed to compare the efficacy of pain relief in patients with knee OA by prescribing a selected group of patients a combination of NSAIDs (naproxen) with duloxetine and the other group NSAIDs with pregabalin and comparing them with patients receiving only NSAIDs. This was to find out the best possible treatment plan for patients suffering from this debilitating and progressive joint disease.

## Materials and methods

This was a single blinded, prospective, randomised study, which was approved by Institutional Research and Ethics Forum, Rawalpindi Medical University (Ref. 620/IREF/RMU/2024), registered with Clinical Trial Unit (CTU) of Rawalpindi Medical University (Ref. CTU/03/2025/003/RMU) and carried out between March 2024 and February 2025 and the patients were followed for a total of 12 weeks of duration. Priori sample size calculation was performed for the determination of an adequate number of participants required to detect a clinically meaningful difference of 1.0 point on the VAS for pain between the treatment groups. Assuming an SD of 1.5, a two-sided alpha level of 0.05, and a desired power of 80%, it was estimated that a minimum of 30 patients per group would be necessary. To account for potential dropouts, 35 patients were initially allocated to each group, resulting in a total of 105 randomised patients. Written consent was obtained via a consent form in Urdu, and either a signature or thumbprint was obtained from the participating patients. Inclusion criteria were age>50 years, OA diagnosed via the American College of Rheumatology criteria [13], baseline moderate pain scores (VAS equal or more than 4, WOMAC score equal or more than 8) and suffering from knee OA pain for at least 14 days during the past three months. Exclusion criteria were the presence of a psychiatric or neurological disorder (which could affect pain assessment), inflammatory arthritis, age<50 years, previous allergy to naproxen, duloxetine or pregabalin, and history of previous knee surgery or traumatic knee injury. Patients fulfilling the inclusion criteria were allocated (1:1:1) via simple randomisation, into one of the three treatment groups: group A (NSAID), group B (NSAID + duloxetine) and group C (NSAID + pregabalin). All patients were on prophylactic proton pump inhibitors to prevent adverse gastrointestinal side effects secondary to naproxen. The randomisation sequence was generated utilising a computer-based random number table. Allocation concealment was ensured using SNOSE (sequentially numbered, opaque, sealed envelopes). Naproxen 500mg twice daily was chosen to be the NSAID of our choice for this study, along with a proton pump inhibitor cover. For group B patients, duloxetine 30mg/day was added to naproxen for the initial four weeks of study and the dose was up-titrated to 60mg/day for the remainder eight weeks. For group C, pregabalin 75mg/day was added on top of naproxen for the first four weeks, which was later escalated to 150mg/day (75mg twice a day) for the remaining eight weeks of the study. The authors involved in data collection were blinded to the treatment protocol given to each patient to reduce observer bias. Patients’ assessment was carried out at baseline (day 0), week 4 and week 12 after the commencement of the treatment protocol. The primary outcome of pain was measured using the VAS and Western Ontario and McMaster Universities Osteoarthritis Index (WOMAC). The secondary outcome was the measurement of depression using the BDI (Beck Depression Inventory) score and assessment of sleep quality using PSQI (Pittsburgh Sleep Quality Index). A one-way ANOVA test (F value) was used to compare the mean across the three groups, followed by Tukey's HSD (honestly significant difference) post hoc test for pairwise comparisons. p-value <0.05 was considered statistically significant.

## Results

A total of 105 patients were inducted into the study after gaining consent and following the inclusion criteria. Thirty-five patients each were randomly allocated into either treatment groups A, B and C. Baseline characteristics including age, gender, baseline VAS, WOMAC, BDI and PSQI scores did not differ significantly between the groups, as confirmed by one-way ANOVA (p>0.05) for all comparisons. Three patients dropped out of group A, five from group B and two from group C before week 4 of assessment. The dropout rate was 8.57% for group A, 14.29% for group B, while it was 5.71% for group C. Since all the patients dropped out before the four-week assessment, they were excluded from the per-protocol analysis, as they contributed no post-baseline data. No imputation was performed for these early dropouts. The detailed data and results are shown in Table 1.

**Table 1:** Mean scores for primary and secondary outcomes Mean scores for pain (VAS, WOMAC), depression (BDI), and sleep quality (PSQI) were measured for all three groups at day 0, week 4 and week 12 of study. VAS: Visual analogue scale; WOMAC: Western Ontario and McMaster Universities Osteoarthritis Index; BDI: Beck Depression Inventory; PSQI: Pittsburgh Sleep Quality Index

Category	Group A (Naproxen)	Group B (Naproxen + Duloxetine)	Group C (Naproxen + Pregabalin)
Total Patients	32	30	33
Mean Age	62.81	64.8	64
Gender (Male)	11	9	8
Gender (Female)	21	21	25
Mean VAS Score (Day 0)	5.62	5.93	5.85
Mean VAS Score (4 Weeks)	4.44	4.63	4.55
Mean VAS Score (12 Weeks)	4.09	3.03	3.12
Mean WOMAC Score (Day 0)	56.84	62.67	59.94
Mean WOMAC Score (4 Weeks)	45.16	47.87	47.7
Mean WOMAC Score (12 Weeks)	42.25	29.8	30.3
Mean BDI Score (Day 0)	13.34	11.43	12.18
Mean BDI Score (4 Weeks)	10.94	9.27	9.88
Mean BDI Score (12 Weeks)	10.28	5.03	5.64
Mean PSQI Score (Day 0)	7.66	7.23	7.09
Mean PSQI Score (4 Weeks)	6.53	6.27	6.55
Mean PSQI Score (12 Weeks)	6.19	3.37	3.42

**Figure 1:**
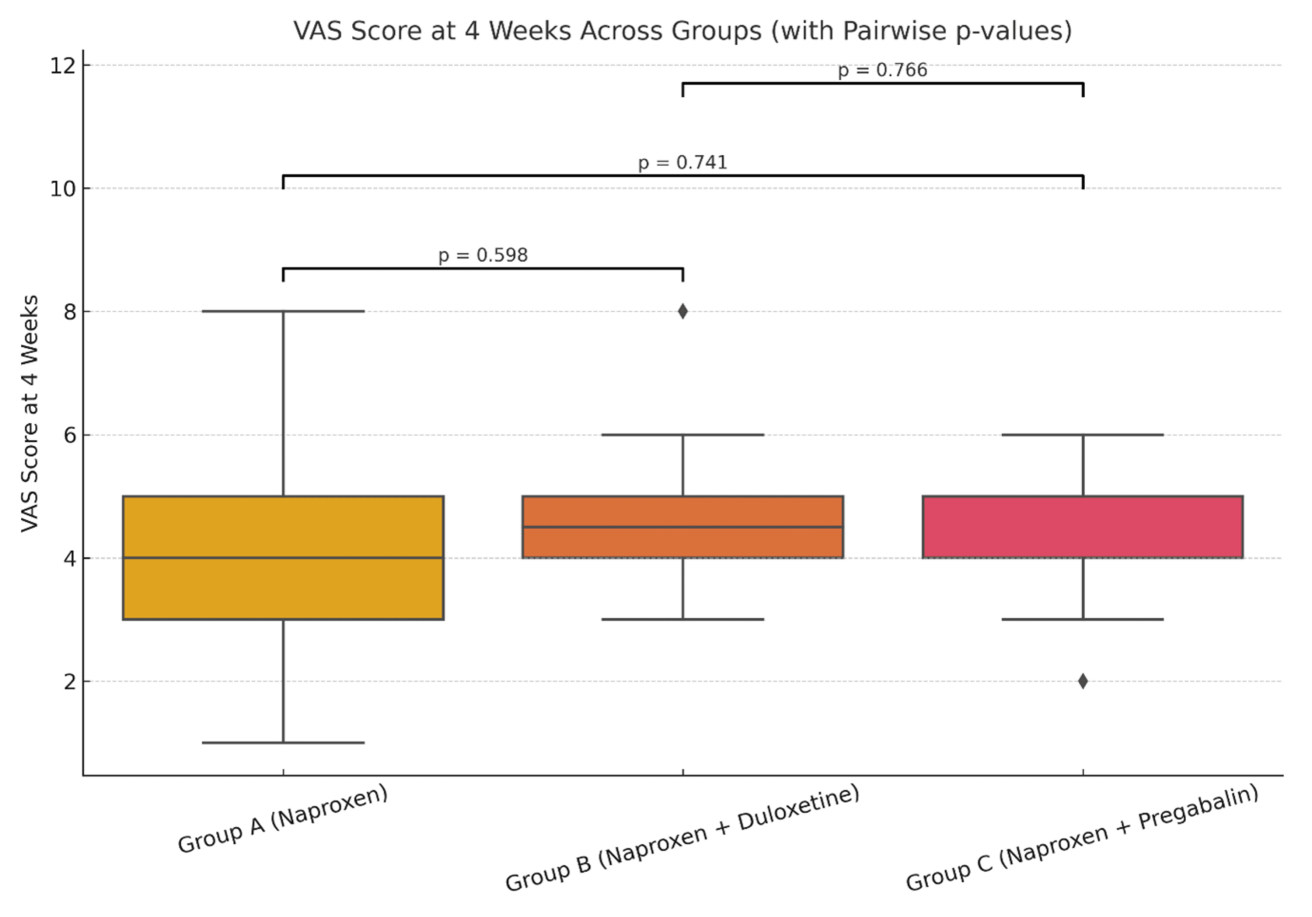
Comparison of the mean VAS score at four weeks The mean VAS score at four weeks was 4.44 for group A, 4.63 for group B and 4.55 for group C (F=0.279, p=0.757). Comparison of the mean VAS score at four weeks between the three groups did not show any statistical significance (p >0.05). VAS: Visual analogue scale

**Figure 2:**
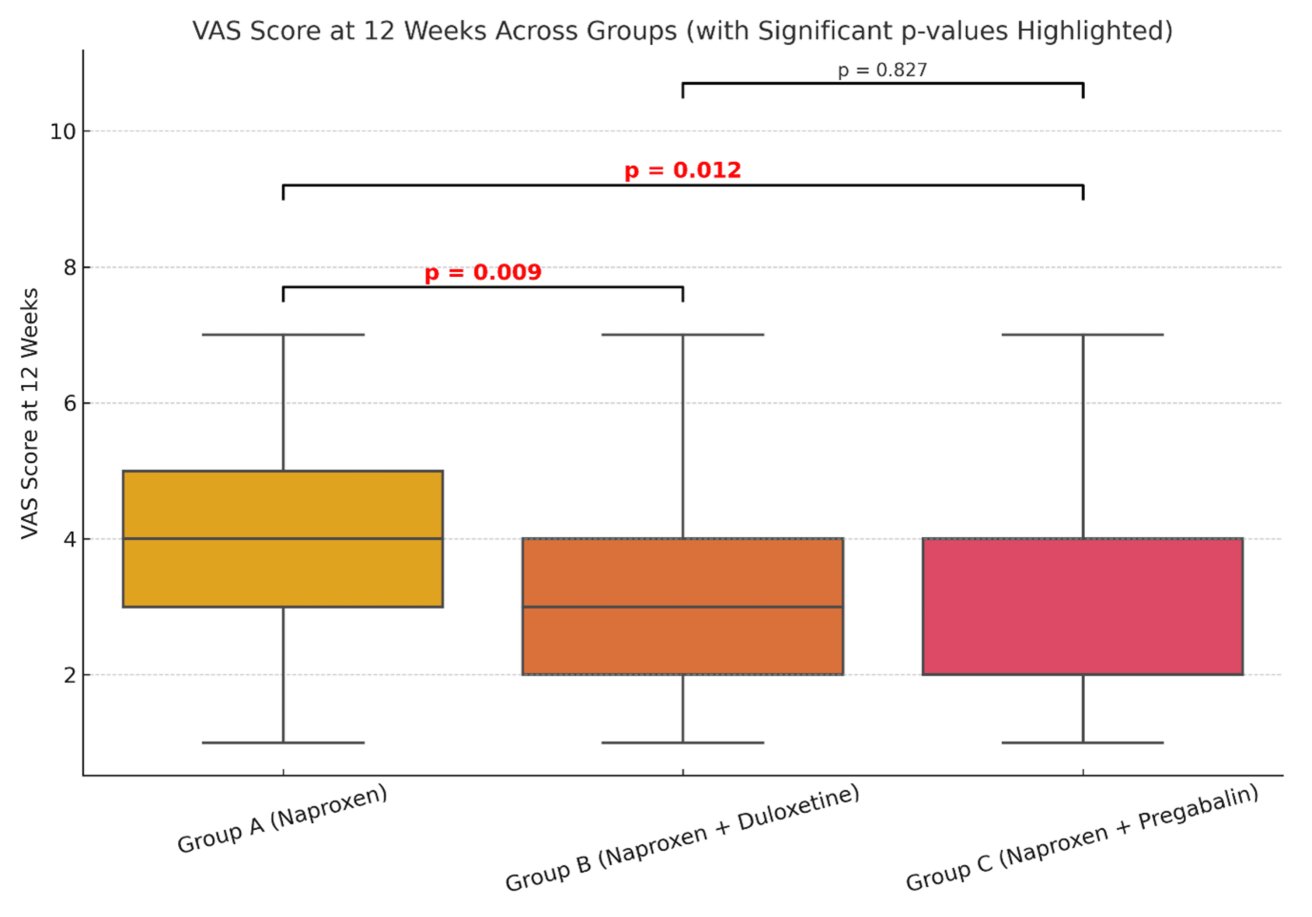
Comparison of the mean VAS score at 12 weeks The mean VAS score at 12 weeks was 4.09 for group A, 3.03 for group B and 3.12 for group C (F=4.175, p=0.0185). Comparison of mean VAS scores at 12 weeks between the three groups showed statistically significantly improved pain scores in group B (p=0.009, 95% CI: 0.28 to 1.84) and group C (p=0.012, 95% CI: 0.21 to 1.73) as compared to group A. There was no statistically significant difference in pain scores between group B and group C (p=0.827). VAS: Visual analogue scale

**Figure 3:**
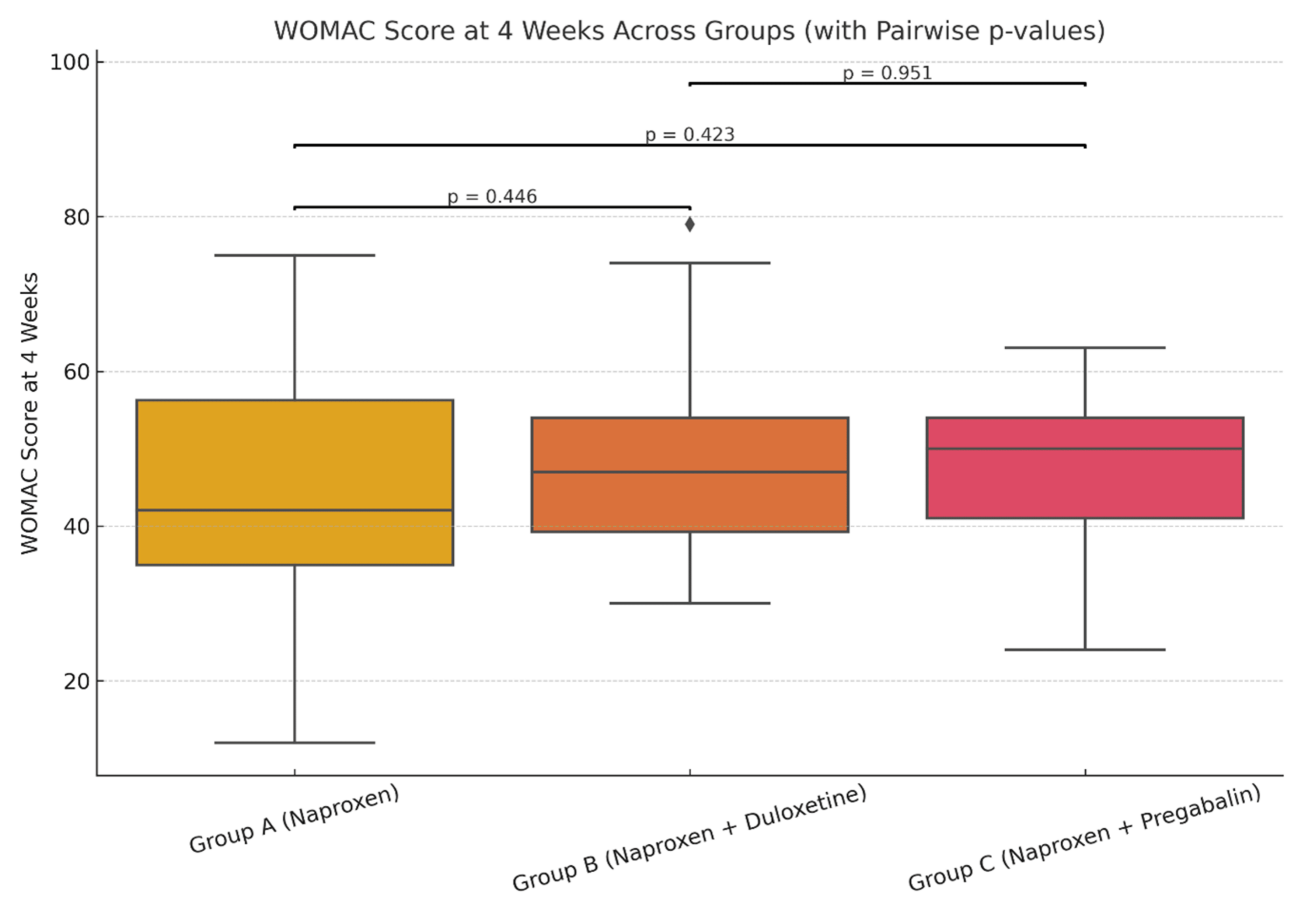
Comparison of the mean WOMAC score at four weeks The mean WOMAC score at four weeks was 45.16 in group A, 47.87 in group B and 47.7 in group C (F=0.615, p=0.543). There was no statistically significant difference in the mean WOMAC scores at four weeks between the three groups (p>0.05). WOMAC: Western Ontario and McMaster Universities Osteoarthritis Index

**Figure 4:**
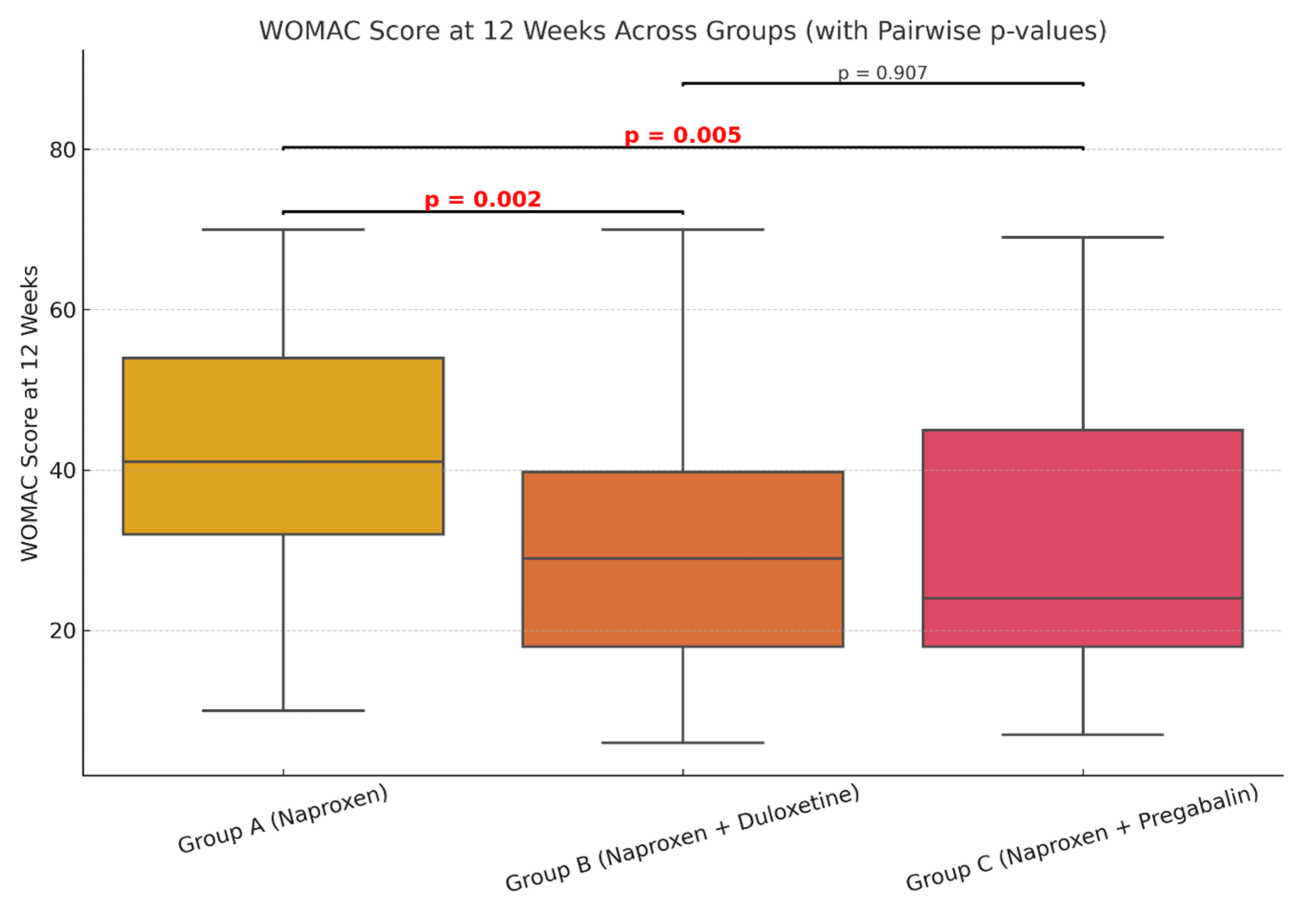
Comparison of the mean WOMAC score at 12 weeks The mean WOMAC score was 42.25 in group A, 29.8 in group B and 30.3 in group C (F=5.627, p=0.005). The mean WOMAC score at 12 weeks was significantly reduced in group B (p=0.002, 95% CI: 7.25 to 17.65) and group C (p=0.005, 95% CI: 6.89 to 17.01) as compared to group A. There was no statistically significant difference when comparing the mean WOMAC scores between group B and group C (p=0.907). WOMAC: Western Ontario and McMaster Universities Osteoarthritis Index

The mean VAS score at four weeks (Table 1) for group A (naproxen only) was 4.44, for group B (naproxen + duloxetine) it was 4.63, while the mean VAS (four weeks) was 4.55 for group C (naproxen + pregabalin). At four weeks, comparison of the mean VAS pain score across the three groups did not show any statistical significance (Figure 1). However, when comparing the mean VAS score at 12 weeks, there was statistically significant improvement (Figure 2) in groups B (p=0.009) and group C (p=0.012) as compared to the naproxen-only group (group A). A similar trend of statistical significance was observed when comparing the WOMAC score at four weeks (Figure 3) and 12 weeks of study (Figure 4), showing that the difference in the mean pain scores was statistically significant only when assessed after 12 weeks of study. The ANOVA test (F-value), followed by Tukey's HSD post hoc test, was used for pairwise comparisons. No statistical significance was detected when comparing either VAS (p=0.827) or WOMAC (p=0.907) at 12 weeks between groups B (naproxen + duloxetine) and group C (naproxen + pregabalin).

**Figure 5:**
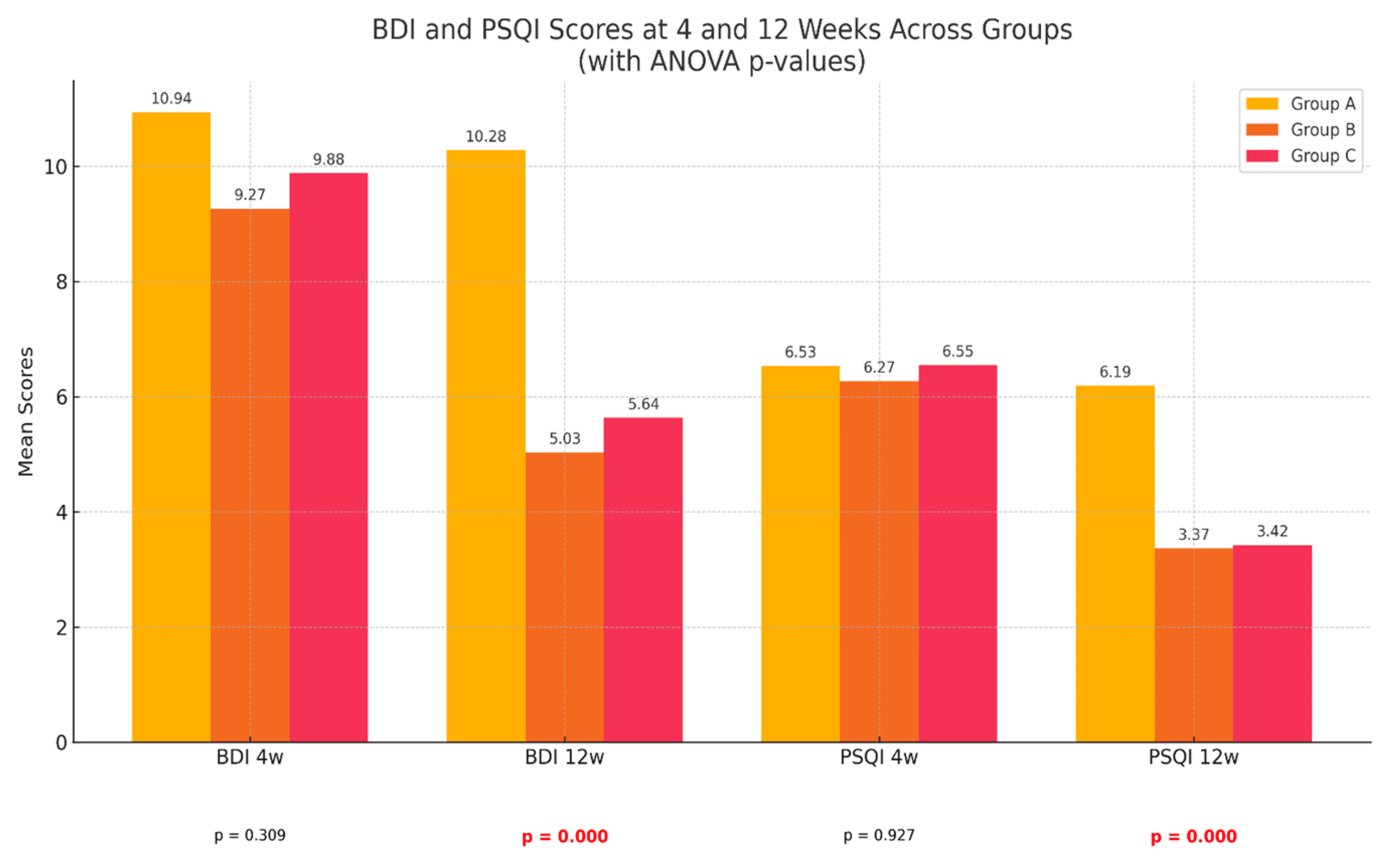
Comparison of mean BDI (depression) and PSQI (sleep quality) scores at four weeks and 12 weeks between three groups Mean BDI and PSQI scores at four weeks and 12 weeks are shown. There was a statistically significant difference in the mean BDI (p<0.05) and mean PSQI (p<0.05) scores at 12 weeks in group B and group C, when compared to group A. However, at four weeks of study only, the difference in the BDI and PSQI scores between the three groups was not statistically significant (p=0.309 and p=0.927, respectively). BDI 4 weeks (F=0.922, p=0.4013), BDI 12 weeks (F=15.25, p=0.000), PSQI 4 weeks (F=0.016, p=0.9843), PSQI 12 weeks (F=8.639, p=0.0004. BDI: Beck Depression Inventory; PSQI: Pittsburgh Sleep Quality Index

For comparison of secondary outcomes including BDI and PSQI scores (Figure 5), similar tests of significance were used.

Mean BDI scores at four weeks were 10.94 in group A, 9.27 in group B and 9.88 in group C with a p-value of 0.309 (statistically insignificant). At 12 weeks, mean BDI scores were significantly lower (p<0.05) in both groups B and C as compared to the naproxen-only group (group A). A similar trend of significance existed when comparing PSQI scores at four weeks and 12 weeks of study, with both groups B (naproxen + duloxetine) and group C (naproxen + pregabalin) showing statistically significant lower scores (p<0.05) only at 12 weeks in comparison to group A (naproxen only). 

With regard to the adverse effects profile, dyspepsia was reported in two patients from group A (6.2%), one patient from group B (3.3%) and two patients from group C (6%). Somnolence was reported in four patients from group B (13.3%), while six patients from group C (18%) reported mild symptoms of dizziness. None of the adverse effects were severe enough to warrant dropping any patient from our study.

## Discussion

Knee joint OA is one of the most prevalent musculoskeletal disorders encountered by both physicians and surgeons worldwide. According to an estimate, knee and hip OA stand at the 11th position on the list of highest contributors to global disability [14]. Due to its progressive and debilitating nature, often the disease requires surgical knee joint transplantation [15], and since this is a disease mostly encountered in the middle-aged and older age population, patients are at risk of post-operative complications [16].

Determining an optimal drug regimen for any chronic disease is always cumbersome, as numerous patient-to-patient factors and variability must be taken into consideration. The purpose of this 12-week study was to try to find out a combination drug regimen that can help the OA patients with their pain as well as increase their overall lifestyle satisfaction, by measuring sleep scores and looking for any signs of depression along with the pain from OA.

Since the data collection was done at three intervals, i.e., day 0, week 4 and week 12, it helped in better understanding the optimal duration needed for assessment of the effect of naproxen, duloxetine and pregabalin on the pain from knee OA. As evident from mean pain scores (VAS, WOMAC) at four weeks across the three groups, it is evident that all the drugs’ regimens influenced the pain scores, albeit modestly. However, when assessed after 12 weeks, the analgesic effect of combination regimens was probably far greater and statistically significant on pain management. This might be explainable due to the prolonged time needed by duloxetine and pregabalin to exert their maximal effects. These results highlighted the fact that if patients suffering from knee OA are commenced on a combination drug regimen involving either naproxen and duloxetine, or naproxen and pregabalin, this can potentially lead to very effective pain management, as the end-study VAS and WOMAC scores at 12 weeks reduced to the category of only mild pain intensity.

Sleep and depression are two of the most important long-term sequels of any chronic disorder [17]. Assessing secondary outcomes of any chronic disorder is important to ensure good compliance of the patients with the long-term therapy. As evident from Figure 5, the BDI and PSQI scores at four weeks across the three groups decreased mildly as compared to the baseline; however, the change had no statistical significance. In contrast, at 12 weeks, there was a statistically significant improvement in depression (BDI scores) and sleep quality (PSQI scores) among group B (naproxen + duloxetine) and group C (naproxen + pregabalin), but not in group A (naproxen only). With improvement in sleep scores and depression index, better patient compliance with medical therapy, and an optimal improvement in quality of life could be ensured in the combination drug therapy groups.

Reference studies [8,12] also assessed pain management in knee OA. However, none of them directly compared the quality of response between combination drug regimens. Also, in our study, secondary outcomes of sleep quality and depression were also assessed at day 0, four weeks and 12 weeks into the study.

Since the study was only limited to 12 weeks duration, to gain a consensus on a world-wide scale, we feel that these results need further investigation on a larger sized population group as well as keeping the follow-up of the patients for prolonged duration, whilst ensuring to keep a check on adverse effect profile [18]. If this combination drug regimen does prove to be significantly better in pain management in future studies, we hope that this would lead to a reduced percentage of patients opting for surgical knee replacements [19] as well as avoiding the prevalence of post-operative complications [20].

## Conclusions

Combination drug regimens involving either naproxen and duloxetine or naproxen and pregabalin probably result in better pain management as well as improvement in sleep quality and depression scores in patients with knee OA. At four weeks, the effectiveness of this combination regimen was not significantly superior as compared to naproxen-only therapy; however, at 12 weeks, there was a significant improvement in pain control as well as better secondary outcomes. Further research with a larger sample size, prolonged duration of study and observing the adverse effect profile would be needed to ensure the consistency of these results.
